# Expression regulation of *MALATE SYNTHASE* involved in glyoxylate cycle during protocorm development in *Phalaenopsis aphrodite* (Orchidaceae)

**DOI:** 10.1038/s41598-020-66932-8

**Published:** 2020-06-22

**Authors:** Wan-Lin Wu, Yu-Yun Hsiao, Hsiang-Chia Lu, Chieh-Kai Liang, Chih-Hsiung Fu, Tian-Hsiang Huang, Ming-Hsiang Chuang, Li-Jun Chen, Zhong-Jian Liu, Wen-Chieh Tsai

**Affiliations:** 1 Shenzhen Key Laboratory for Orchid Conservation and Utilization, The National Orchid Conservation Center of China and The Orchid Conservation & Research Center of Shenzhen, Shenzhen, 518114 China; 2 Institute of Tropical Plant Sciences and Microbiology, National Cheng Kung University, Tainan, Taiwan; 3 Orchid Research and Development Center, National Cheng Kung University, Tainan, Taiwan; 4 Key Laboratory of National Forestry and Grassland Administration for Orchid Conservation and Utilization at College of Landscape Architecture, College of Landscape Architecture, Fujian Agriculture and Forestry University, Fuzhou, China; 5 Fujian Colleges and Universities Engineering Research Institute of Conservation and Utilization of Natural Bioresources, College of Forestry, Fujian Agriculture and Forestry University, Fuzhou, 350002 China; 6 Department of Life Sciences, National Cheng Kung University, Tainan, 701 Taiwan; 7 Department of Electrical Engineering, National Cheng Kung University, Tainan, 701 Taiwan; 8 Center for Big Data Research, Kaohsiung Medical University, Kaohsiung, Taiwan

**Keywords:** Plant embryogenesis, Plant molecular biology

## Abstract

Orchid (Orchidaceae) is one of the largest families in angiosperms and presents exceptional diversity in lifestyle. Their unique reproductive characteristics of orchid are attracted by scientist for centuries. One of the synapomorphies of orchid plants is that their seeds do not contain endosperm. Lipids are used as major energy storage in orchid seeds. However, regulation and mobilization of lipid usage during early seedling (protocorm) stage of orchid is not understood. In this study, we compared transcriptomes from developing *Phalaenopsis aphrodite* protocorms grown on 1/2-strength MS medium with sucrose. The expression of *P. aphrodite MALATE SYNTHASE* (*PaMLS*), involved in the glyoxylate cycle, was significantly decreased from 4 days after incubation (DAI) to 7 DAI. On real-time RT-PCR, both *P. aphrodite ISOCITRATE LYASE* (*PaICL*) and *PaMLS* were down-regulated during protocorm development and suppressed by sucrose treatment. In addition, several genes encoding transcription factors regulating *PaMLS* expression were identified. A gene encoding homeobox transcription factor (named *PaHB5*) was involved in positive regulation of *PaMLS*. This study showed that sucrose regulates the glyoxylate cycle during orchid protocorm development in asymbiotic germination and provides new insights into the transcription factors involved in the regulation of malate synthase expression.

## Introduction

**Figure 1:**
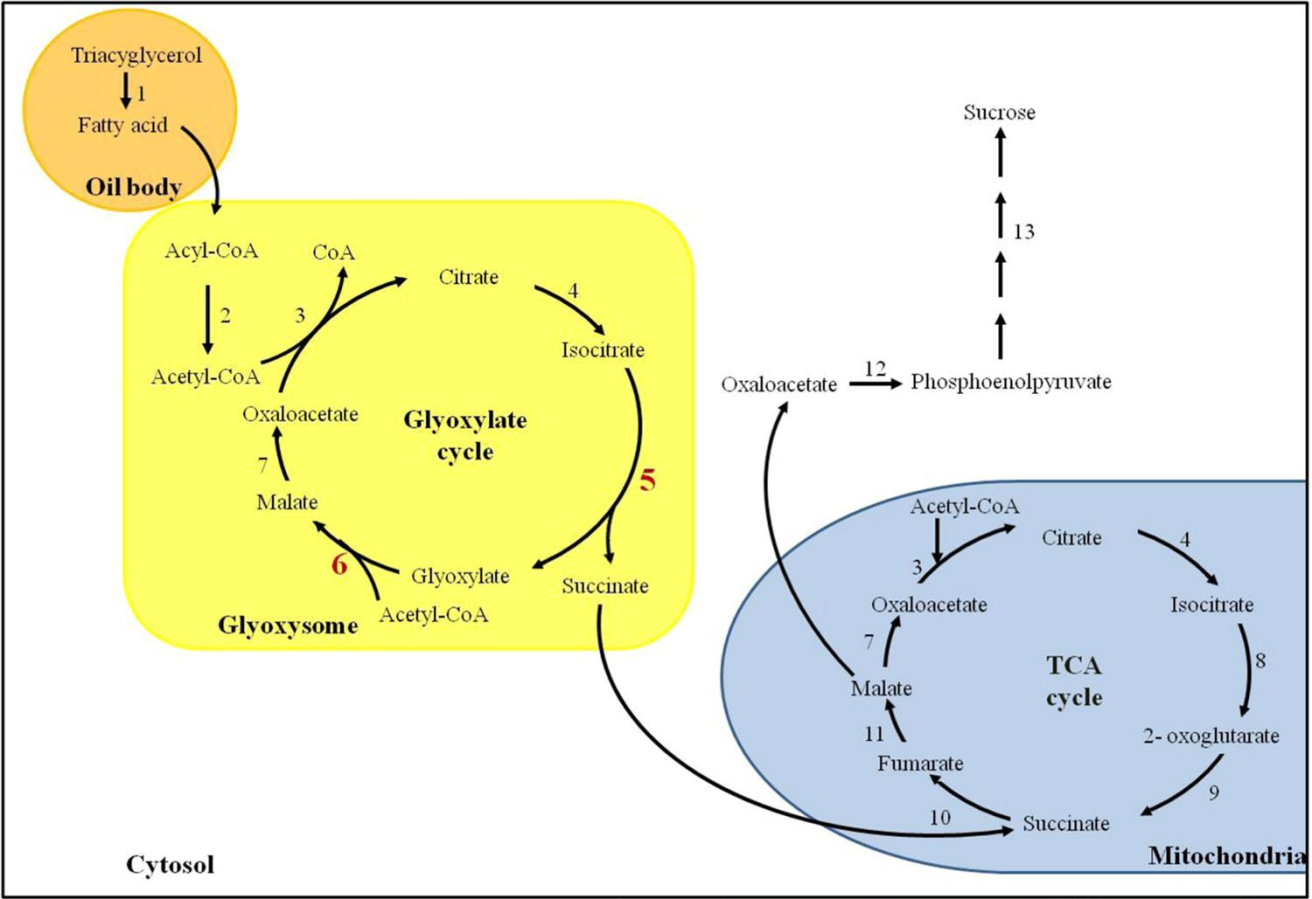
Oil mobilization pathway in oil seeds. 1, triacylglycerol lipase; 2, fatty acid β-oxidation; 3, citrate synthase; 4, aconitate hydratase; 5, isocitrate lyase; 6, malate synthase; 7, malate dehydrogenase; 8, isocitrate dehydrogenase; 9, 2-oxoglutarate dehydrogenase and succinyl-CoA synthetase; 10, succinate dehydrogenase; 11, fumarate hydratase; 12, phosphoenolpyruvate carboxykinase; 13, gluconeogenesis.

In many dicot seeds, such as in *Arabidopsis thaliana*, *Glycine max*, and *Brassica napus*, a large amount of lipids is stored in cotyledons, which are therefore called “oil seeds”. During oil seed germination, lipids cannot be directly used by seeds to provide energy. Therefore, the oil mobilization pathway, including β-oxidation, glyoxylate cycle, tricarboxylic acid (TCA) cycle and gluconeogenesis, is essential for converting storage lipids to soluble sugars that are used to fuel seedling growth (Fig. 1). In oil mobilization, the glyoxylate cycle plays a central role in use of seed storage oil. The cycle uptakes the two-carbon product acetyl-CoA and synthesizes the 4-carbon compound succinate, which subsequently forms a substrate for gluconegenesis to generate sucrose in the cytosol. These soluble sugars are then catabolized by glycolysis to support seedling growth^1,2^. The glyoxylate cycle involves two unique key enzymes: isocitrate lyase (ICL, EC 4.1.3.1), which catalyzes the lysis of isocitrate to glyoxylate and succinate, and malate synthase (MLS, EC 2.3.3.9), which synthesizes malate from glyoxylate and acetyl-CoA^1,2^. In Arabidopsis seedlings, the transcript level and enzyme activity of ICL and MLS are induced after imbibition and decrease after post-germinative growth. This expression pattern is strongly correlated with lipid breakdown^1^. Control of plant metabolism at the transcription level is most apparent when the intracellular nutritional status changes. In the regulation of the glyoxylate cycle, carbon catabolite repression has been well studied in cell culture, seedlings and mature plant tissue of cucumber^3–5^. The gene expression of *ICL* and *MLS* was induced in cucumber cell culture during sugar starvation^1^ and was down-regulated on treatment with different hexose sugars (sucrose, glucose, fructose and mannose). Deletion analysis of the promoter region of cucumber *ICL* and *MLS* revealed separate conserved sequence elements that are necessary for induction in response to a change in carbohydrate status^3^. Suppressed ICL enzyme activity was also found in the presence of glucose in Arabidopsis during post-germinative growth^6^.

The seeds of orchid are often referred to as ‘dust seeds. The seed is very tiny and contains a globular-shape embryo without well development as in other flowering plants^7,8^. The seed development in orchids is unique as compared with most flowering plants. Once the ovules mature, a zygote and a polar chalazal complex could be formed after successful double fertilization^7^. However, the polar chalazal complex do not has ability to develop into an endosperm^9^. Thus, the mature orchid seeds do not contain endosperm. The lipid is the major energy storage found in orchid embryo.

Because of the minute size and limited stored nutrient reserves of orchid seeds, symbiosis with mycorrhizal fungus under natural conditions is essential for germination. Seed reserves are mobilized to provide nutrition for early seedling development before photosynthesis^7,8^. After the fungus is established in the orchid, growth generally occurs with carbon flux from fungi. The swollen embryos grow and form protocorms, a structure between the embryo and the seedling that lacks chlorophyll^8^. After germination, young seedlings have insufficient reserves to allow for ongoing growth without fungus-supplied carbon^10^. On realizing that the main function of the fungus is to provide a carbon source, sugar was added into culture medium to enable asymbiotic germination^8^. A wide range of sugars has been shown to support germination and growth of orchid seeds; they include mono-, di- and oligosaccharides (such as glucose, fructose, maltose and trehalose)^11–13^. This technique of asymbiotic orchid seed germination is useful for propagation for most orchids in the absence of mycorrhizal fungus^7^. However, how the carbon source affects the metabolic events of orchid seed storage oil during protocorm development remain the least studied and most poorly understood.

In this study, we compared the expression of both *P. aphrodite ISOCITRATE LYASE* (*PaICL*) and *PaMLS* genes regulated by sucrose at protocorm stage. We identified a positive transcription factor (TF) regulating *PaMLS* expression, thereby providing the basis for an expanded understanding of orchid seed storage-oil utilization.

## Results

### Distribution of storage products in *P. aphrodite* mature seeds

**Figure 2:**
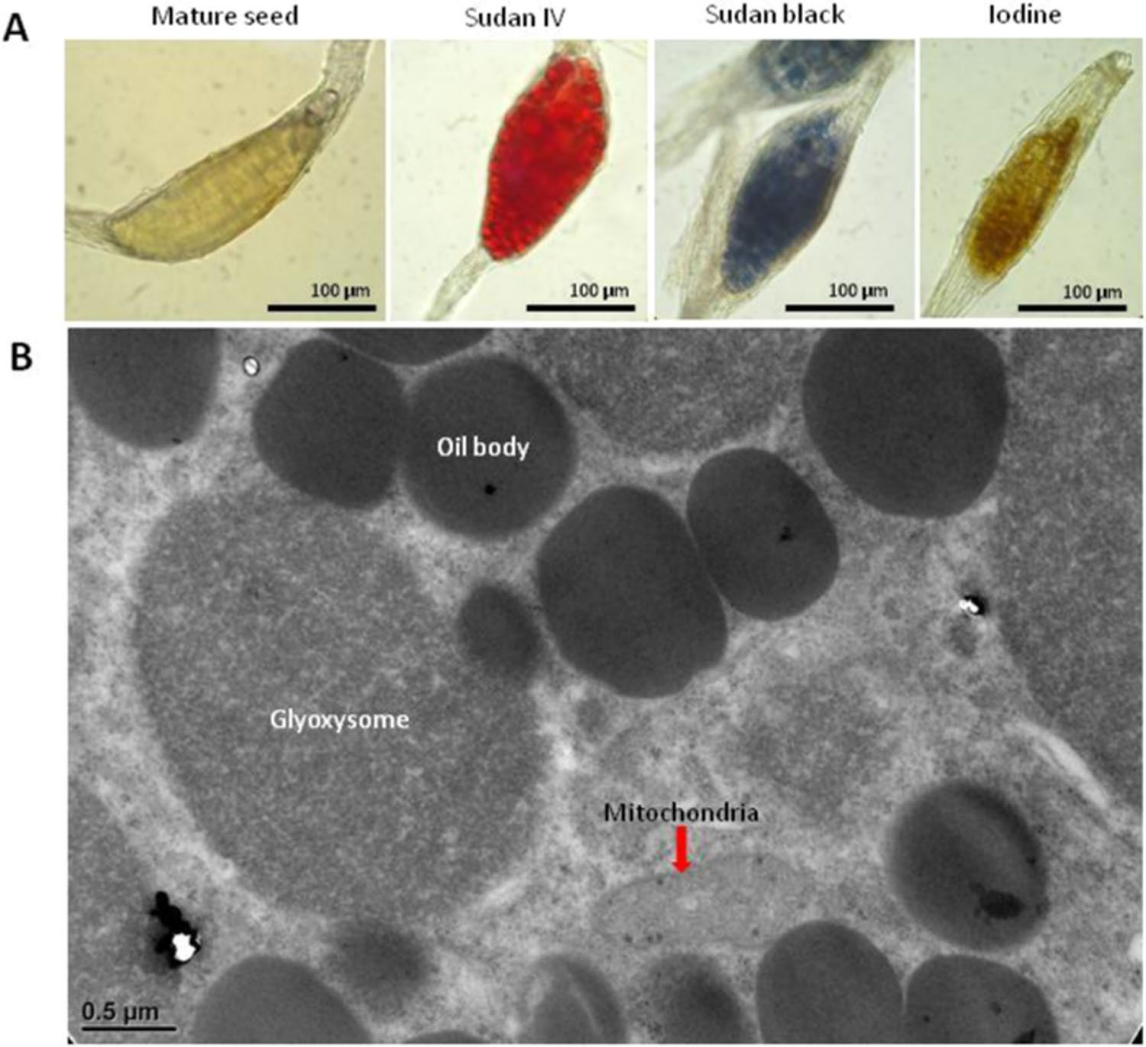
Distribution of storage components in *Phalaenopsis aphrodite* mature seeds (**A**) *P. aphrodite* mature seeds and seeds stained with Sudan IV, Sudan black and iodine. Large amounts of lipids were visualized in red with Sudan IV and in dark-blue with Sudan black. Absence of starch deposition is seen as dark-brown on iodine staining. (**B**) Transmission electron micrograph of a section of a *P. aphrodite* protocorm at 0 DAI. DAI, days after incubation.

According to previous study, endosperm is absent in mature orchid seeds, but lipids are accumulated during the seed maturation stage. To verify the storage components in *P. aphrodite* mature seed, we used 0.3% Sudan IV solution and 0.3% Sudan black solution to stain for lipids (Fig. 2A). The entire proembryo of seeds was red with Sudan IV staining and dark-blue with Sudan black staining. Therefore, a large amount of lipids accumulated in the proembryo of mature *P. aphrodite* seed. However, the absence of starch was visualized as dark-brown staining with 5% iodine solution.

### TEM of a section in a *P. aphrodite* protocorm at 0 DAI

In germinating seed, fatty acids are released from lipids stored in oil bodies, then imported into glyoxysomes to produce succinate via β-oxidation and the glyoxylate cycle. Succinate molecules are then transported to mitochondria to be converted to malate for gluconeogenesis. Therefore, the location of oil bodies, glyoxysomes and mitochondria are close in germinating seeds. To investigate the spatial distribution of the three organelles in germinating orchid seeds, *P. aphrodite* seeds were resuspended in 1/2 MS liquid medium to imbibe the seeds (0 DAI protocorms) and observed by TEM (Fig. 2B). Many oil bodies were found in protocorms at 0 DAI, shown in dark-grey, with glyoxysomes (light-grey organelles) located beside them. Mitochondria were in close proximity to glyoxysomes and oil bodies.

### Effect of sucrose on protocorm development in *P. aphrodite*

**Figure 3:**
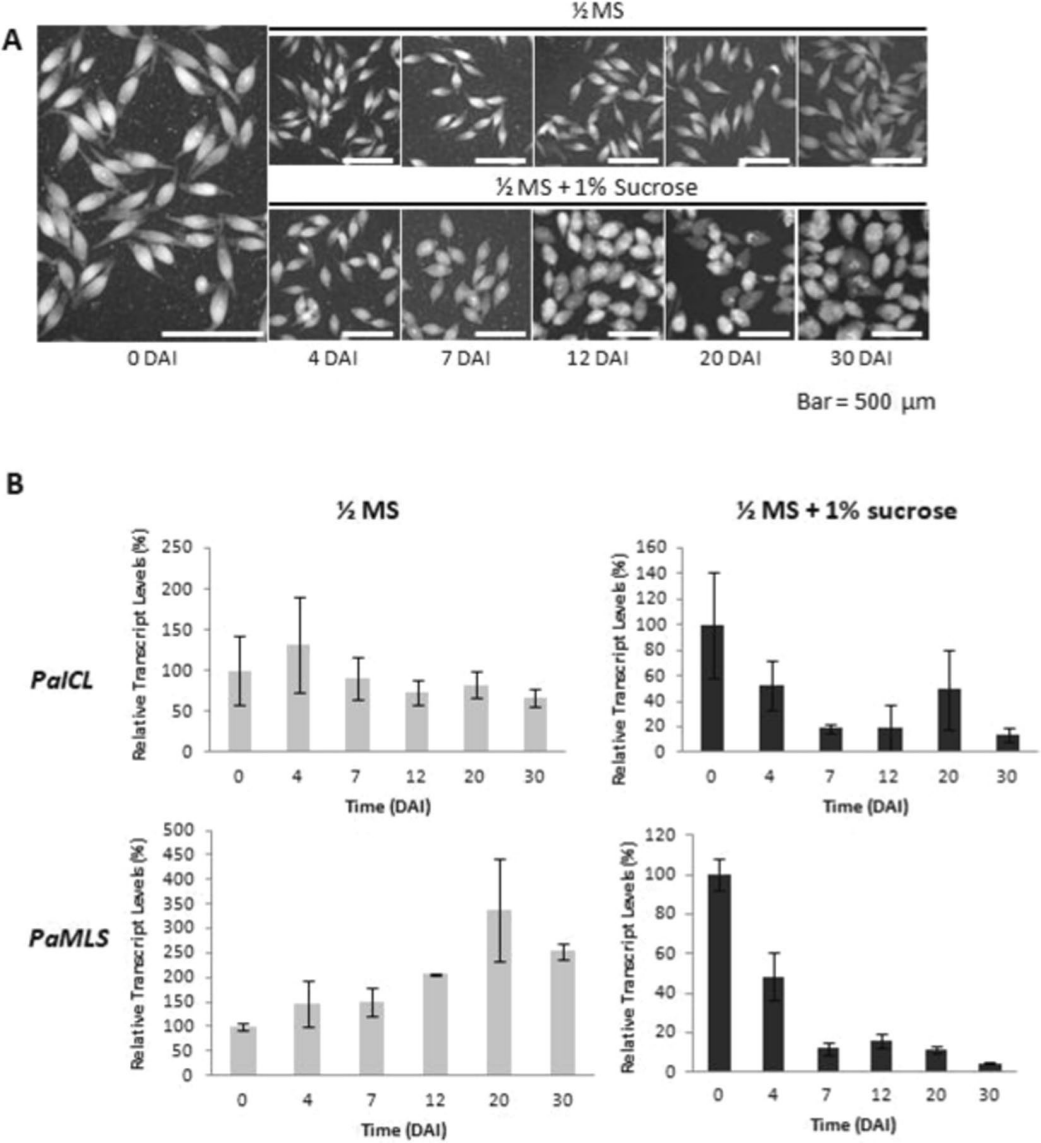
Phenotypes and expression profiles of *PaICL and PaMLS* at different developmental protocorm stages in *P. Aphrodite* (**A**) *P. aphrodite* seeds were resuspended in 1/2-strength MS (1/2 MS) liquid medium for 48 h, defined as 0-DAI protocorms, then incubated in 1/2 MS medium with or without 1% sucrose for 4 to 30 days. DAI, days after incubation. Bar = 500 μm. (**B**) Real-time quantitative RT-PCR analysis of *PaICL* and *PaMLS* expression normalized to *Phalaenopsis Actin4* for each sample; experiments were performed in triplicate. Data are mean ± SEM.

To examine the effect of sucrose on protocorm development, *P. aphrodite* mature seeds were grown in medium containing 1/2-strength MS salts with or without 1% (w/v) sucrose (Fig. 3A). In the presence of sucrose, spindle-like seeds swelled and greenish protocorms were observed at 7 DAI. Most protocorms were greenish and became round and a few protocorms were bleached from 12 to 20 DAI. At 30 DAI, many protocorms were bleached and some continue enlarged and turned to dark green. In contrast, seeds grown without sucrose did not swell and turn greenish until 30 DAI. These results suggest that sucrose is important to provide energy for supporting orchid protocorm development and/or germination.

### Expression of genes involved in oil mobilization pathway

**Figure 4:**
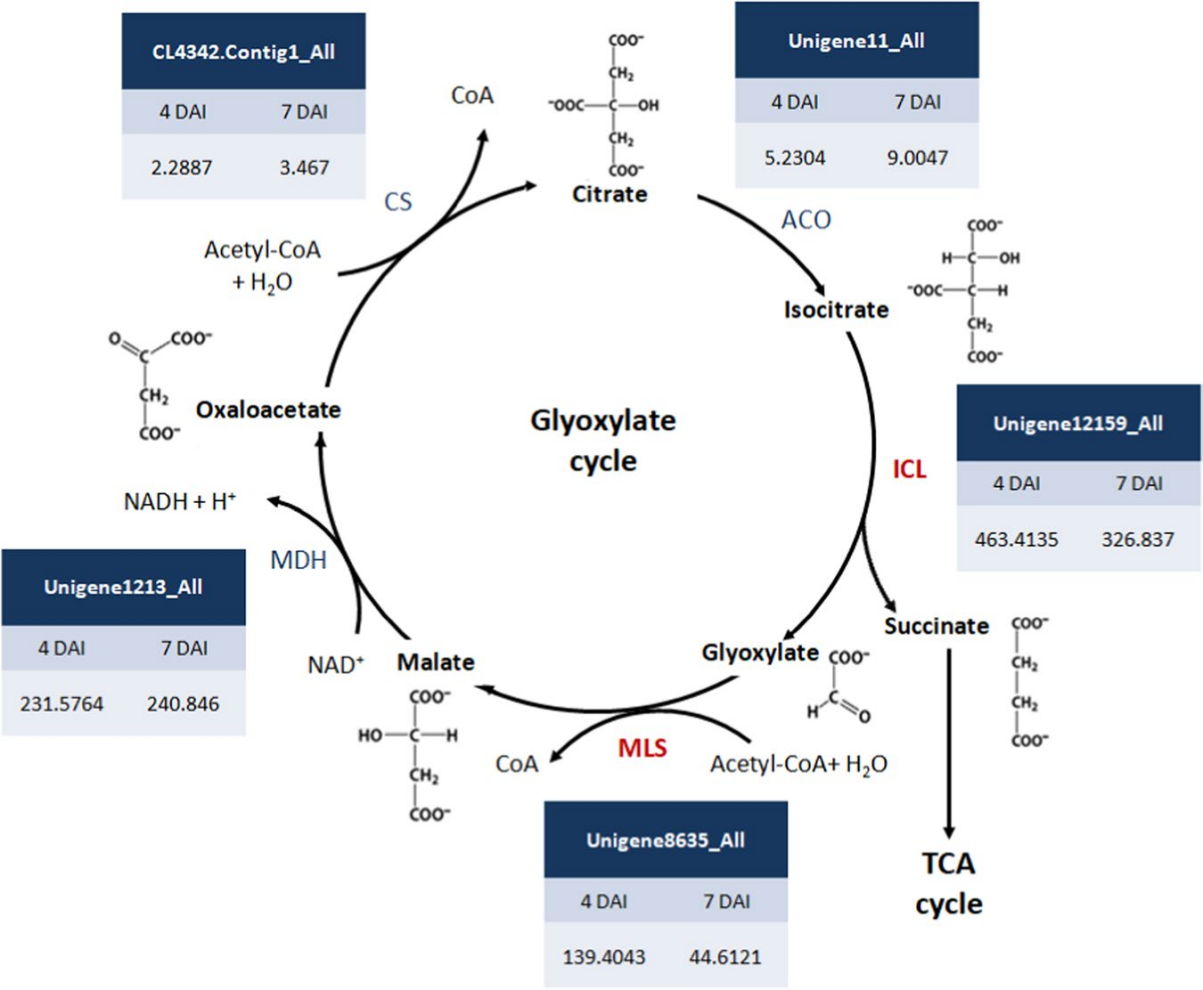
Expression of unigenes involved in glyoxylate cycle in 4- and 7-DAI protocorms The expression levels were derived from *P. aphrodite* protocorm transcriptomes. ACO: aconitate hydratase, ICL: isocitrate lyase, MLS: malate synthase, MDH: malate dehydrogenase, CS: citrate synthase.

**Figure 5:**
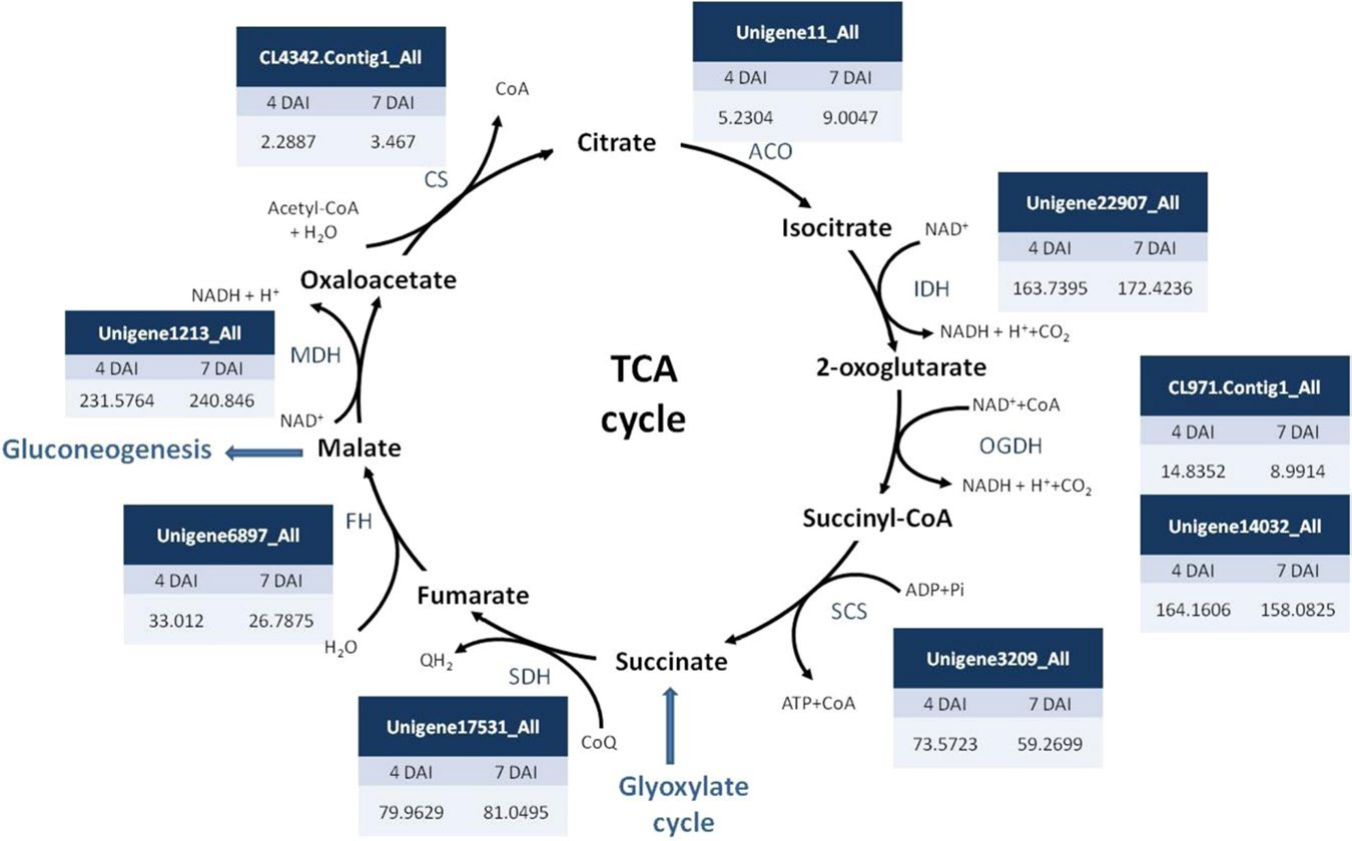
Expression of unigenes involved in tricarboxylic acid (TCA) cycle between 4- and 7-DAI protocorms. The expression levels were derived from *P. aphrodite* protocorm transcriptomes. CS: citrate synthase, ACO: aconitate hydratase, IDH: isocitrate dehydrogenase, OGDH: 2-oxoglutarate dehydrogenase, SCS: succinyl-CoA synthetase, SDH: succinate dehydrogenase, FH: fumarate hydratase, MDH: malate dehydrogenase.

**Figure 6:**
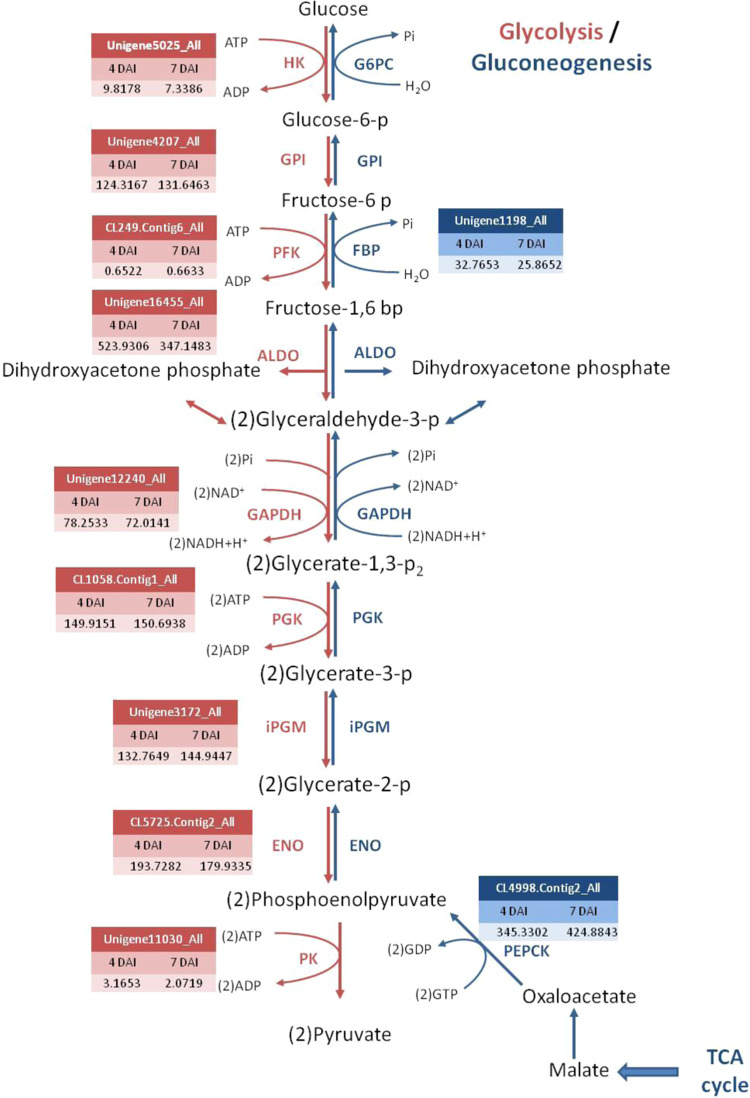
Expression of unigenes involved in glycolysis/gluconeogenesis between 4- and 7-DAI protocorms. The expression levels were derived from *P. aphrodite* protocorm transcriptomes. HK: hexokinase, G6PC: glucose-6-phosphatase, GPI: glucose-6-phosphate isomerase, PFK: 6-phosphofructokinase 1, FBPase: fructose-1,6-bisphosphatase I, ALDO: fructose-bisphosphate aldolase, GAPDH: glyceraldehydes 3-phosphate dehydrogenase, PGK: phosphoglycerate kinase, iPGM: 2,3-bisphosphoglycerate-independent phosphoglycerate mutase, ENO, enolase, PK: pyruvate kinase, PEPCK: phosphoenolpyruvate carboxykinase.

The expression of *ICL* and *MLS* involved in the glyoxylate cycle has been found correlated with lipid breakdown and regulated by the hexose sugars^1^. To further reveal whether sucrose affects the protocorm oil mobilization pathway, we compared transcriptomes from 4- and 7-DAI protocorms treated with sucrose. The expression of enzymes involved in the glyoxylate and TCA cycles and gluconeogenesis and glycolysis pathway were compared by differential expression analysis. Unigenes involved in each pathway were obtained by using genes sequences from the KEGG Pathway (http://www.genome.jp/kegg/pathway.html ^14^) to blast against protocorm transcriptomes. The expression levels were shown with FPKM in 4- and 7-DAI protocorm transcriptomes. In the glyoxylate cycle, *MLS* showed 3-fold down-regulation and *ICL* 1.4-fold down-regulation in 7-DAI protocorms. We found no significant changes in unigene hits for other enzymes (Fig. 4). However, sucrose did not significantly affect the expression of genes in the TCA cycle (Fig. 5) or gluconeogenesis and glycolysis pathway (Fig. 6): the expression of unigenes involved in these pathways did not significantly differ between 4- and 7-DAI protocorms. Interestingly, hexokinase, phosphofrucokinase, and pyruvate kinase genes involved in glycolysis showed low FPKM value at both 4 and 7 DAI.

### Identification and phylogenetic analysis of *PaICL* and *PaMLS* in *P. aphrodite*

**Figure 7:**
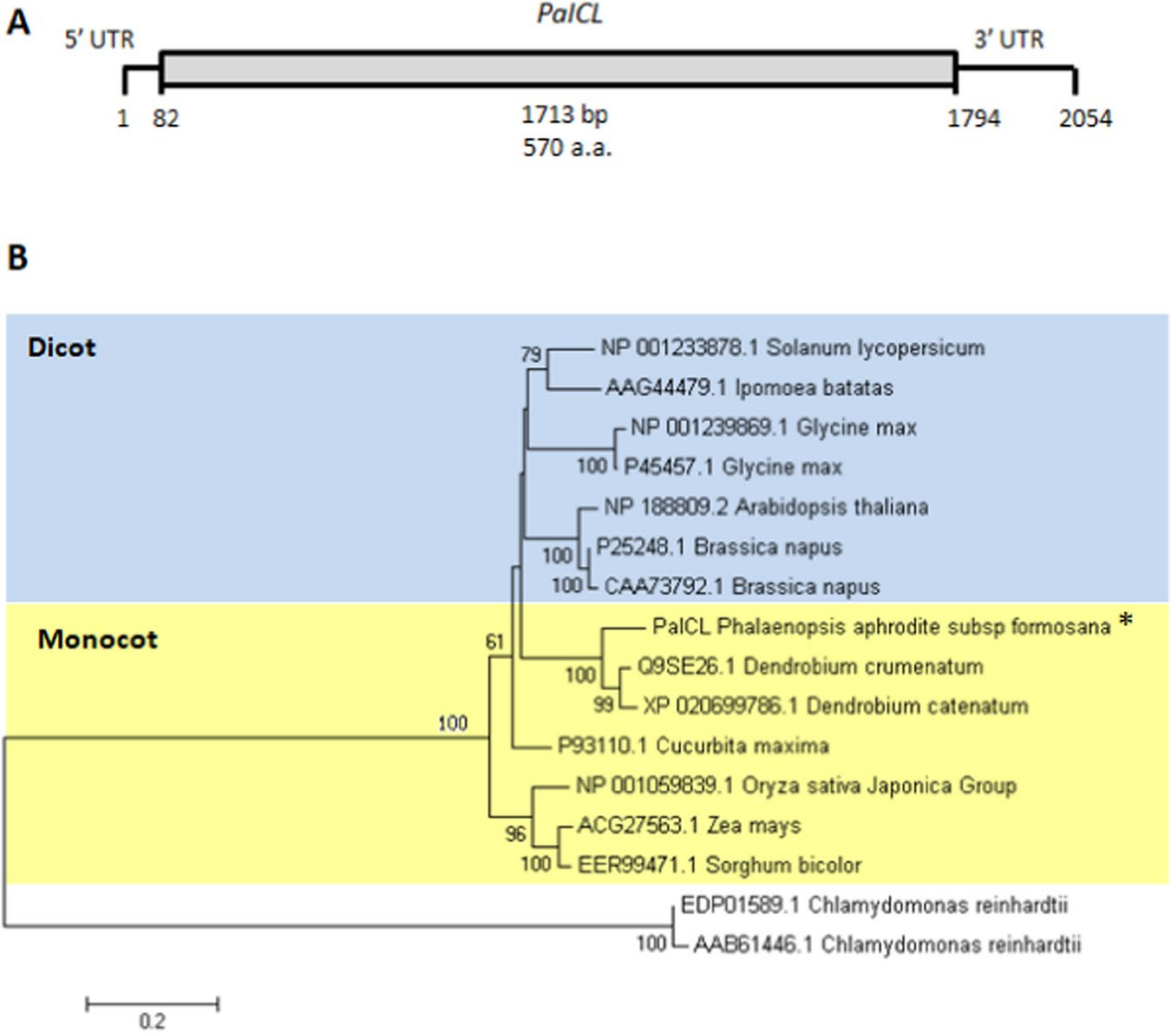
Phylogenetic analysis of *ICL*. (**A**) Structure of *PaICL* full-length cDNA. (**B**) Phylogenetic tree created with MEGA 5.0 by the neighbor-joining method and the bootstrap test involved 1000 iterations.

**Figure 8:**
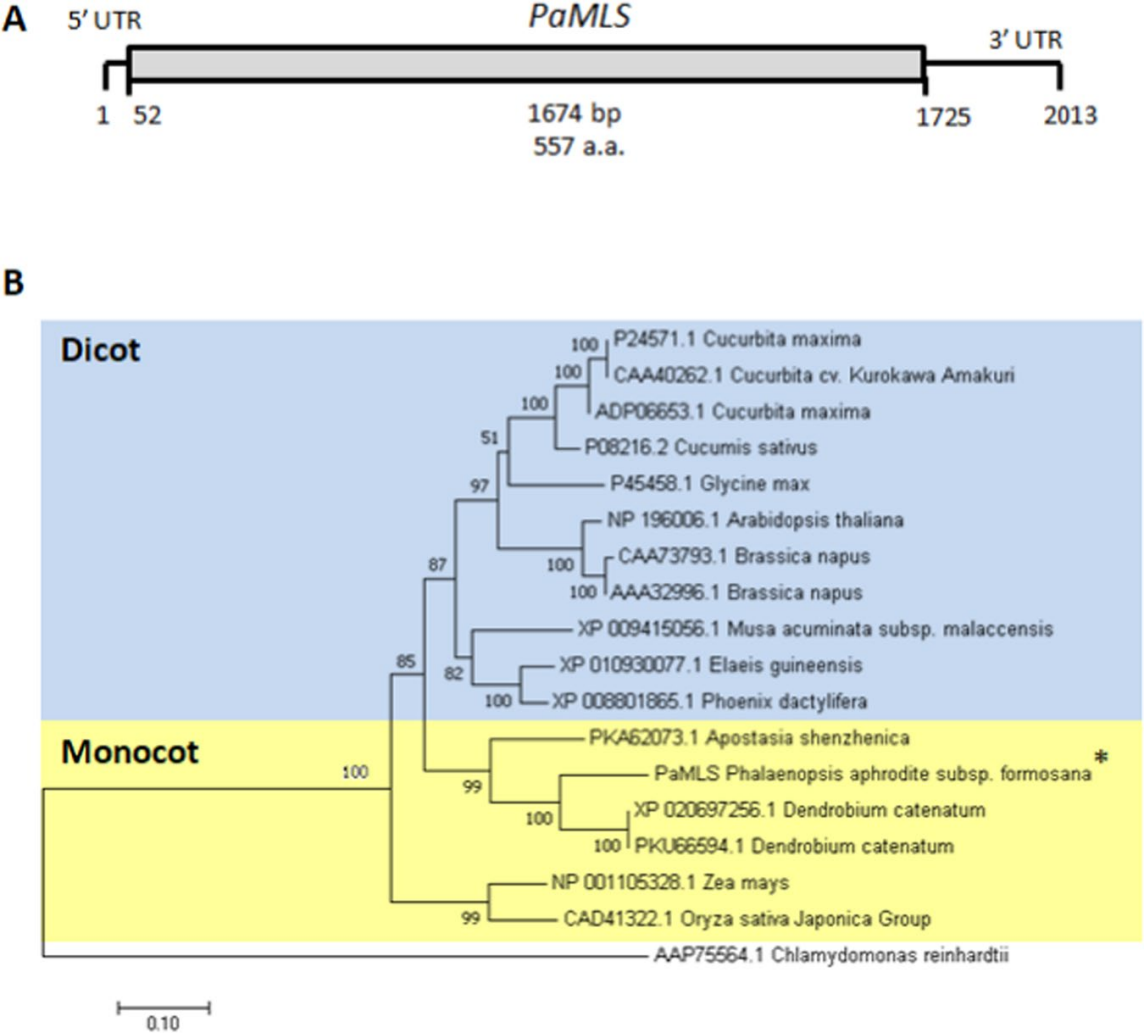
Phylogenetic analysis of *MLS*. (**A**) Structure of of *PaMLS* full-length cDNA. (**B**) Phylogenetic tree created with MEGA 5.0 by the neighbor-joining method and the bootstrap test involved 1000 iterations.

Full-length cDNA sequences of *P. aphrodite* ICL and MLS were obtained from protocorm cDNA at 0 DAI by 5′ RACE and were named *PaICL* and *PaMLS*, respectively. Full-length cDNA of *PaICL* was 2,054 bp with the coding region from 82 to 1,794 bp (Fig. 7A). Full-length cDNA of *PaMLS* was 2,013 bp with the coding region from 52 to 1,725 bp (Fig. 8A). Conceptual translation of the open reading frames encoded by genes yielded proteins 570 and 557 amino acids long for *PaICL* and *PaMLS*, respectively.

To determine the phylogenetic relationships of *PaICL* and *PaMLS* with other plant isocitrate-lyase and malate-synthase genes, we reconstructed the phylogenetic tree for these genes by using the coding regions of amino acid sequences retrieved from the National Center for Biotechnology Information (NCBI). Both PaICL (Fig. 7B) and PaMLS (Fig. 8B) were closely related to their respective orthologs in the primitive orchid *Apostaisa*^15^ and the other epiphytic orchid *Dendrobium*^16^. Both PaICL and PaMLS formed a clade with the homologs of monocots (Figs. 7B and 8B).

### Temporal expression of *PaICL* and *PaMLS* during protocorm development

To verify the effects of sucrose on glyoxylate cycle during protocorm development in *P. aphrodite*, we used real-time quantitative PCR to confirm the temporal expression patterns of *PaICL* and *PaMLS* (Fig. 3B). Without sucrose in the medium, *PaICL* and *PaMLS* expressed in the entire protocorm developmental stages, from 0 to 30 DAI. In contrast, with sucrose, *PaICL* and *PaMLS* were substantially down-regulated during protocorm development. Thus, sucrose might inhibit lipid hydrolysis through the glyoxylate cycle during orchid protocorm development.

### Comparison of sucrose-affected transcript profiles between protocorms at 4 and 7 DAI

To gain more insight into the genes related to the decreased expression of *PaMLS* in 7-DAI protocorms treated with sucrose, we used a 2-fold expression cutoff for selecting differentially expressed unigenes between protocorms at 4 and 7 DAI. In total, 3,830 and 7,648 genes showed differential expression in protocorms at 4 and 7 DAI, respectively.

**Figure 9:**
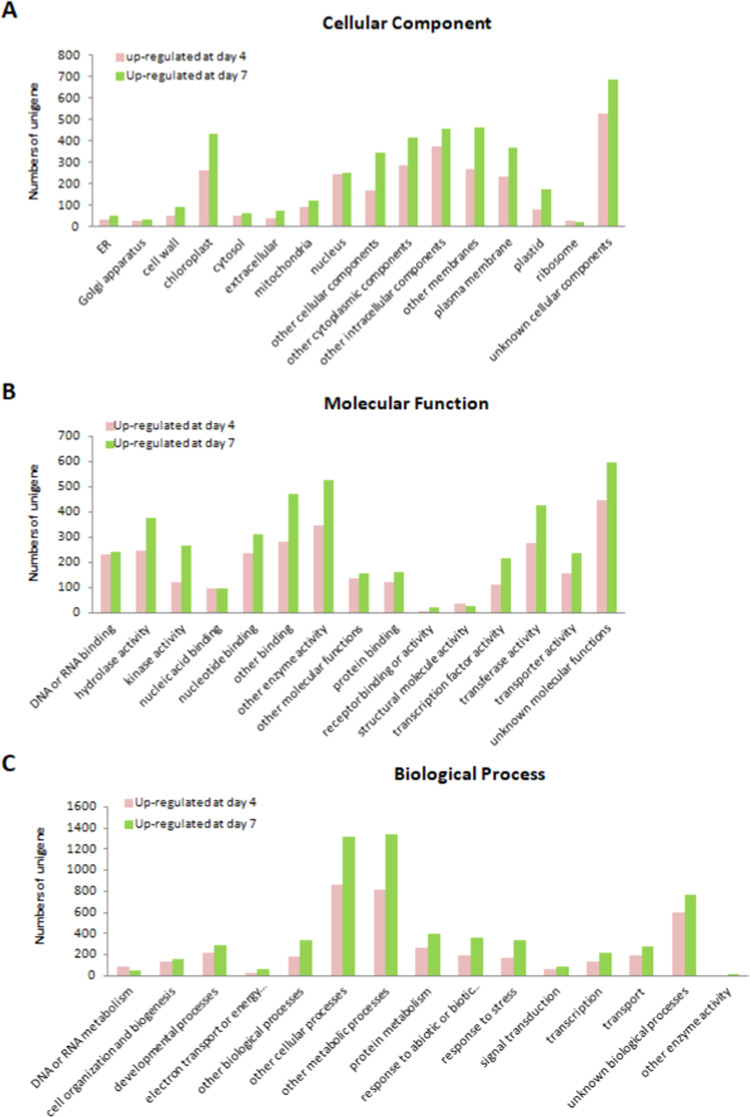
Gene ontology (GO) assignments for 4- and 7-DAI dominant genes derived from *P. aphrodite* protocorm transcriptomes. Genes were filtered with absolute value of log_2_ ratio ≧1. The GO Slim Classification for Plants developed at TAIR was used to characterize the unigenes functionally. (**A**) Cellular component, (**B**) molecular function, (**C**) biological process.

Gene Ontology (GO) classification system for plants developed at TAIR^17^ (https://www.arabidopsis.org/help/helppages/go_slim_help.jsp) was further adopted to characterize the possible function of developing stage-dominant unigenes (Fig. 9). For 4-DAI protocorm-dominant unigenes, 90% (3,451/3,830), 65% (2,510/3,830), 64% (2,467/3,830) were partitioned to biological processes, molecular functions and cellular components, respectively, and for 7-DAI protocorm-dominant unigenes, 72% (5,513/7,648), 49% (3,796/7,648), 49% (3,798/7,648) were assigned, respectively. The 7-DAI protocorm unigenes were highly represented in most GO categories. In biological processes, 7-DAI protocorm unigenes were involved in other cellular processes and other metabolic processes, but 4-DAI protocorm unigenes were involved in DNA or RNA metabolism. In molecular functions, 7 DAI protocorm unigenes were highly represented in hydrolase activity, kinase activity and TF activity, but 4-DAI protocorm unigenes were involved in nucleic acid binding and structural molecule activity. In cellular components, 7-DAI protocorm unigenes were overrepresented in chloroplasts, other cellular components and plastids as compared with 4-DAI protocorm unigenes. In contrast, 7-DAI protocorm unigenes were slightly underrepresented in ribosomes.

### Characterization of developing stage–dominant unigenes by KEGG pathway analysis

Stage–dominant unigenes were mapped to KEGG pathways (http://www.genome.jp/kegg/pathway.html) (Table 1). About 10.2% (392/3,830) of 4-DAI protocorm-dominant unigenes were mapped to 93 KEGG pathways, and 8.6% (656/7,648) of 7-DAI unigenes were mapped to 97 KEGG pathways. Among 392 hits of 4-DAI protocorm-dominant unigenes, 251 hits were assigned to metabolism, 104 to genetic information processing, 17 to organismal systems, 16 to cellular processes, and 4 to environmental information processing. 656 7-DAI protocorm-dominant unigenes were respectively classified into metabolism (521), genetic information processing (73), organismal systems (41), cellular processes (18), and environmental information processing (3). In metabolism, the number of unigenes associated with biosynthesis of other secondary metabolites, carbohydrate metabolism, and lipid metabolism was obviously increased in 7-DAI protocorms. In lipid metabolism, more genes were involved in α-linolenic acid metabolism in 7- than 4-DAI protocorms, which suggests that α-linolenic acids might be the main components in orchid seed-storage lipids. The significant increase in biosynthesis of secondary-metabolite genes indicated that protocorms may generate secondary metabolites to adapt to the environment.

**Table 1:** Number of differentially expressed unigenes mapped in KEGG pathways.

KEGG pathway	Sub-pathway of KEGG pathway	No. of hits for 4-DAI protocorm unigenes	No. of hits for 7-DAI protocorm unigenes
**Metabolism**		**251**	**521**
	Carbohydrate Metabolism	50	105
	Energy Metabolism	7	50
	Lipid Metabolism	27	75
	Nucleotide Metabolism	30	14
	Amino Acid Metabolism	46	79
	Metabolism of Other Amino Acids	10	25
	Glycan Biosynthesis and Metabolism	7	18
	Metabolism of Cofactors and Vitamins	23	19
	Metabolism of Terpenoids and Polyketides	27	44
	Biosynthesis of Other Secondary Metabolites	24	92
**Genetic Information Processing**		**104**	**73**
	Transcription	21	13
	Translation	17	13
	Folding, Sorting and Degradation	24	32
	Replication and Repair	42	15
**Environmental Information Processing**		**4**	**3**
	Signal Transduction	4	3
**Cellular Processes**		**16**	**18**
	Transport and Catabolism	16	18
**Organismal Systems**		**17**	**41**
	Environmental Adaptation	17	41

### Transcription factors related to the regulation of *PaMLS* expression

To explore the TFs related to the regulation of *PaMLS* expression, unigenes with 10-fold differential expression between 4- and 7-DAI protocorms were screened and BLAST searched against rice TFs derived from plantTFDB (Supplementary Fig. S2). Furthermore, 2,000-bp upstream sequences of *PaMLS* translation start sits for the *Phalaenopsis* genome were retrieved^18^ and analyzed by using plantPAN to predict TF binding sites. In all, 14 unigenes with at least 10-fold differential expression and presenting a putative binding sequence at the PaMLS promoter were filtered. Eight genes corresponded to the 14 unigenes after alignment to the *Phalaenopsis* genome (Table 2). These genes were named *PaHB5, PaANT, PaMADS2, PaMYB4, PaPIF3, PaRAV1-1, PaWRKY18* and *PaWRKY71*.

**Table 2:** Identified putative transcription factors for *PaMLS* expression.

Transcription factor	Unigene	Stage with higher expression	Fold change expression	Full length cDNA (bp)	Coding sequence (bp)	Protein length (a.a)
PaHB5	CL2645.Contig2_All	4 DAI	10.5	816	60–686	208
PaWRKY71	CL696.Contig6_All	7 DAI	10.2	990	158–739	192
PaPIF3	Unigene23033_All	7 DAI	10.5	1161	446–751	100
PaANT	Unigene4788_All	7 DAI	14.4	950	42–827	260
PaMADS2	Unigene23959_All	7 DAI	20.4	764	81–764	237
PaMYB4	Unigene23199_All	7 DAI	21.4	702	1–702	232
PaWRKY18	Unigene14862_All	7 DAI	49.2	661	26–538	171
PaRAV1–1	Unigene22378_All	7 DAI	42.8	579	1–579	191

### Temporal expression of 8 putative transcription factors during protocorm development

**Figure 10:**
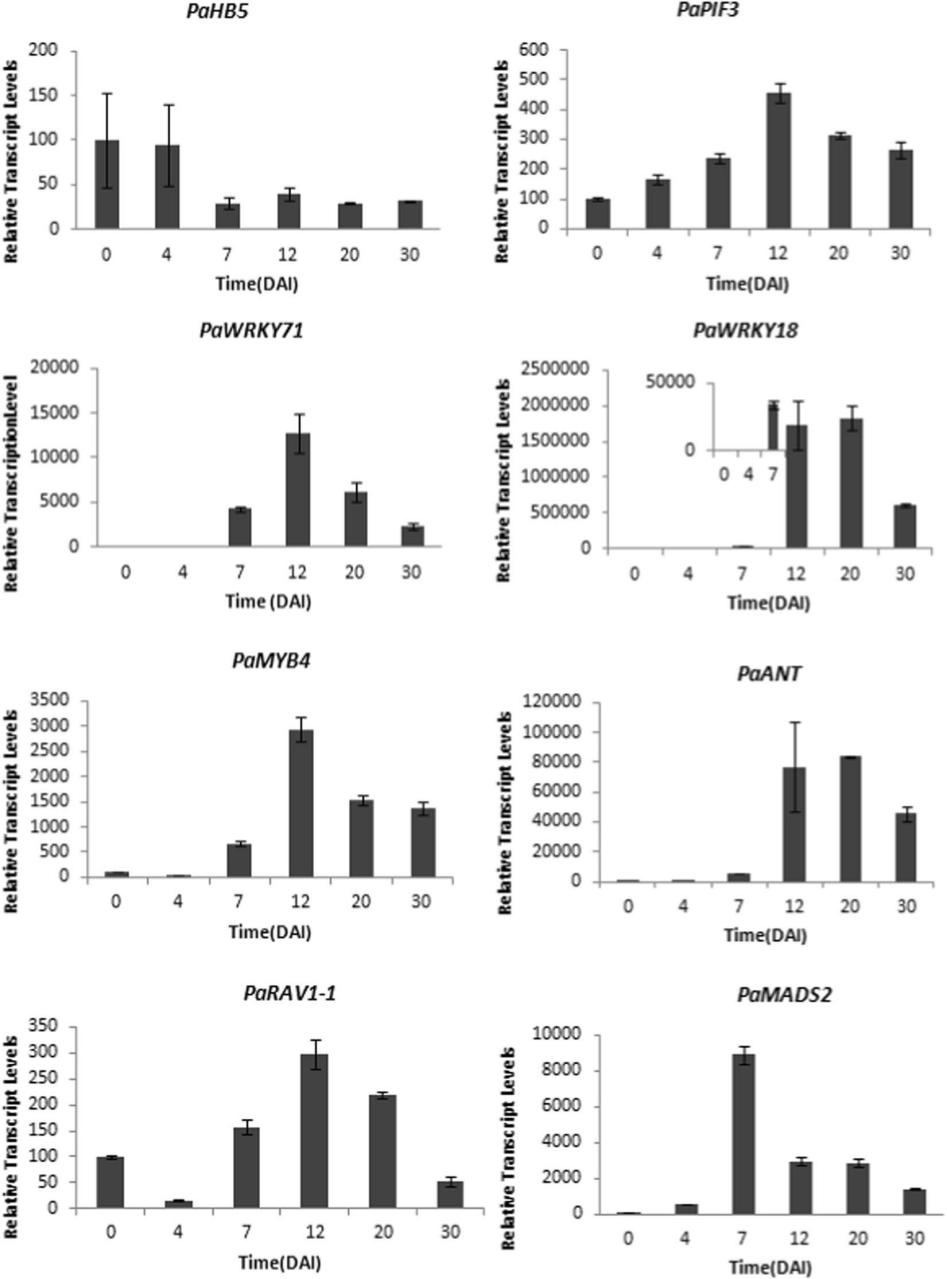
Real-time quantitative PCR analysis of putative transcription factor genes during *P. aphrodite* protocorm development. RNA was extracted from 0- to 30-DAI protocorms cultured with 1% sucrose. Quantification was normalized to *Phalaenopsis Actin4* for each sample; experiments were performed in triplicate. Data are mean ± SEM.

Real-time quantitative PCR were used to confirm the temporal expression of 8 putative TFs during developing protocorms treated with sucrose (Fig. 10). The predicted TF *PaHB5* had higher expression at early protocorm developing stages (0 DAI and 4 DAI), and its expression was significantly decreased from 7 DAI. *PaPIF3* transcripts continually increased and peaked at 12 DAI, then decreased to 30 DAI. *PaWRKY18*, *PaWRKY71*, *PaMYB4*, and *PaANT* showed a similar expression pattern but the expression was extremely low in 0- and 4-DAI protocorms. The expression patterns of *PaRAV1-1* and *PaPIF3* were similar, but *PaRAV1-1* level was decreased in 4-DAI protocorms. *PaMADS2* showed low expression in 0- and 4-DAI protocorms, and highly accumulated transcripts in 7-DAI protocorms, which was earlier than for other putative regulators.

### Activation ability of PaHB5 on *PaMLS* promoter

**Figure 11:**
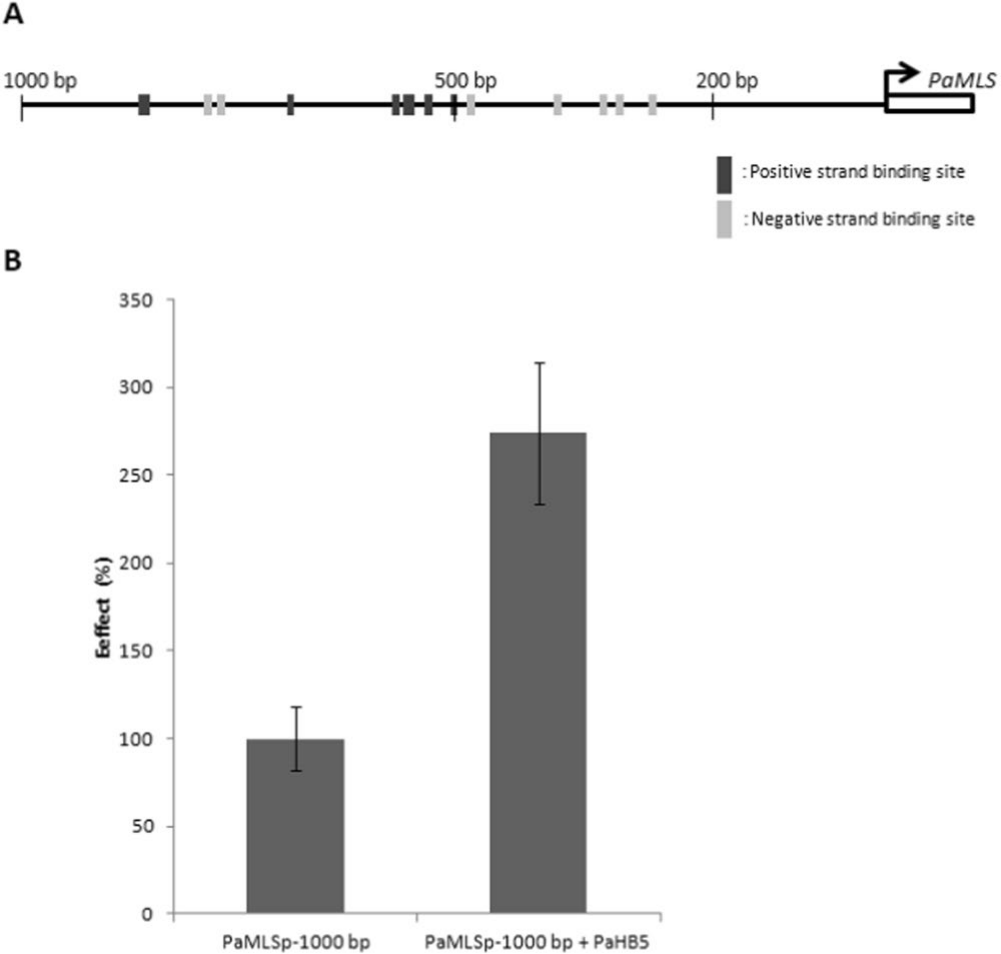
Activation ability of PaHB5 on the *PaMLS* promoter. (**A**) Diagram of 1-kb *PaMLS* promoter. Numbers represent the distance in nucleotides from the 5′ end of the promoter to the start codon of *PaMLS*. The dark-grey and light-grey blocks on the promoter represent the putative binding sites of ATHB5 predicted by promoter analysis with PlantPAN. (**B**) Effect of PaHB5 on *PaMLS* promoter shown by relative luciferase activity of different treatments.

To predict the putative PaHB5 binding sites, a 2,000-bp upstream sequence of *PaMLS* was analyzed by using the plantPAN database, with 22 putative binding sites obtained for PaHB5. Most binding sites were localized in the region of the 1,000-bp upstream regulatory sequence of *PaMLS* (19 binding sites) (Supplementary Table S2). Therefore, a 1-kb fragment of the upstream regulatory sequence of *PaMLS* was selected for dual luciferase assay (Fig. 11A). PaHB5 conferred 2.73-fold transactivation activity on the 1-kb promoter of *PaMLS* (Fig. 11B), which suggests that PaHB5 is a positive regulator of *PaMLS*.

## Discussion

In the life cycle of angiospermss, seed germination and post-germinative growth are pivotal stages^19^. Previous studies have indicated that the glyoxylate cycle plays a crucial role in mobilization of storage oils during germination and post-germinative growth of oilseed^20^. In germinating oilseeds, glyoxylate cycle is located in the glyoxysome surrounded by a specialized single membrane^1^. In this paper, we found a large amount of lipids accumulated in the proembryo of mature *P. aphrodite* seeds while starch is absence (Fig. 2A). We also observed that glyoxysomes exist in the embryo cells of imbibed *Phalaenopsis* orchid seed. In addition, oil bodies and mitochondria could be found in close proximity to glyoxysomes (Fig. 2B). From the observation, it might imply that endogenous reserves of lipid could be used in the imbibed *Phalaenopsis* orchid seed. Interestingly, it has been reported that no glyoxysomes were found in *Catteleya aurantiaca* protocorms grown on medium with sucrose or without sucrose^21^. Another observation from some terrestrial orchids such as *Disa*, *Disperis* and *Huttonaea* indicated that glyoxysomes are absent in seeds incubated without exogenous sucrose, and glyoxysomes appear in the presence of sucrose^22^. The differences could be due to the situations that different species are studied. In fact, previous studies showed that all the glyoxylate cycle’s enzymes are located in the glyoxysome, with the exception of aconitase^1,23^. Detection of significant expression of *PaICL* and *PaMLS* supported that existence of glyoxysomes in *Phalaenopsis* imbibed seeds.

With sucrose in the medium, the expression of both *PaICL* and *PaMLS* was significantly down-regulated from 0 to 7 DAI during protocorm development (Fig. 3B). The expression of both *PaICL* and *PaMLS* may be sensitive markers of exogenous carbon source in orchid seed germination. It has been demonstrated that the fungal requirement of orchid seeds can be successfully bypassed *in vitro* with provision of sucrose in the culture medium^24^. These results suggested that sucrose is a key regulatory factor supplied by the symbiont for regulation of orchid seed lipid metabolism in natural environment. We deduced that at natural condition, if an orchid seed do not meet an appropriate symbiont, it do not geminate and slowly breaks down lipid for longer longevity in the wild. If an orchid seed meet an appropriate symbiont, it will use carbon source provided by the symbiont to geminate and suppress lipid usage. Thus, the minus-sucrose treatment in the medium could be considered similar to seeds which fail to seek out appropriate symbionts. Furthermore, plus-sucrose condition might be similar to the successful symbiotic seed germination in the natural environment.

In oilseeds or cucumber cell culture, the sugar-mediated repression of transcript level or enzyme activity is consistent for both ICL and MLS^4,25^. In *Arabidopsis*, lipid breakdown and hypocotyl elongation were strongly inhibited when Arabidopsis *icl* was grown in the dark, and the *icl* mutant is also deficient in conversion of ^14^C acetate into sugar. In *mls* mutants, inhibition of growth is less severe, and some carbon can be converted from acetate into sugar^20^. Therefore, the glyoxylate produced by ICL may be channeled into gluconeogenesis via the serine–glycine shuttle^26^, which was confirmed by the accumulation of serine and glycine residues in the *mls* mutant^27^. In culture of orchid asymbiotic seeds, sucrose provides the carbon source to protocorms and therefore might mediate the end-product inhibition of the glyoxylate cycle to block lipid breakdown. The repressed expression of PaICL and PaMLS by sucrose during protocorm development might serve to reserve the endogenous energy resource and give priority to utilize the exogenous available carbon source.

Eight putative TFs are predicted to bind on the *PaMLS* promoter, including one putative activator (PaHB5) and 7 putative repressors (PaWRKY71, PaPIF3, PaANT, PaMADS2, PaMYB4, PaWRKY18 and PaRAV1-1) (Table 2). The transcriptional activator PaHB5 showed positive transactivation activity on the *PaMLS* promoter, which suggests that PaHB5 has an important role in regulating the glyoxylate cycle for energy management during protocorm development. Moreover, several putative repressors responding to sucrose were identified to possibly regulate the expression of *PaMLS*. Thus, positive and negative regulation may coordinate the expression of *PaMLS*.

Previous studies showed one CArG box-like motif (CCA/T_6_GG) on the promoter of *MLS* in cucumber^4^. In addition, the expression of *MLS* has been detected in cucumber petal^5^. These results suggest that a floral homeotic MADS TF might have a regulatory role in the expression of *MLS* in cucumber. A number of MADS box proteins were identified as involved in proteome changes of *Oncidium sphacelatum* mycorrhizal protocorms at different developmental stages^28^. In this study, a MADS box TF, PaMADS2, a homolog of PeMADS2, which is involved in sepal and petal development in *P. equestris*^29,30^, was also predicted to suppress *PaMLS* expression (Table 2).

The MADS-box genes may directly control *MLS* expression through the sucrose signaling pathway during protocorm development. MYB and WRKY TFs have been reported to be involved in the sugar response. Two crucial TFs, MYBS1 and MYBGA, were found to integrate diverse nutrient-starvation and gibberellin signaling pathways during germination of cereal grains. Although MYBS1 synthesis is repressed by sugar but induced by sugar starvation in rice, MYB TFs were involved in the metabolic response^31^. Some MYB TFs were found to be glucose-inducible in *Arabidopsis* seedlings^32^. Several WRKY proteins responded to and were up-regulated by sugar starvation^33,34^. For example, the WRKY TF, SUSIBA2, is reported to bind to sugar-responsive elements of the isoamylase 1 promoter in crops^35^. Therefore, the putative MYB and WRKY TFs are potential candidates to regulate *PaMLS* transcription under metabolic changes during orchid protocorm development. Phytochrome-interacting factors, belonging to the Arabidopsis basic helix-loop-helix superfamily^36^, were reported to repress seed germination, promote seedling skotomorphogenesis and promote shade-avoidance by regulating the expression of thousands of genes. They are also required for sucrose-dependent growth promotion during post-germinative growth^37^. Thus, showing a negative correlation with the expression of *PaMLS* in response to sucrose treatment (Fig. 10), PaPIF3 may play a negative regulating role on the repression of *PaMLS* expression. In conclusion, this study provides the basis for understanding the regulation of the use of orchid seed-storage energy.

## Conclusion

We observed that the glyoxysomes locate in close proximity to oil bodies and mitochondria in imbibed *Phalaenopsis* protocorms, suggesting storage oil could be catabolized to provide nutrition for the protocorm development and growth. The expression of the *PaICL* and *PaMLS* involved in glyoxylate cycle could be down-regulated by the exogenous sucrose. The transcriptional activator PaHB5 identified from transcriptome comparison presented positive transactivation activity on the *PaMLS* promoter, which indicates that *PaHB5* play an important role in regulating the glyoxylate cycle for energy management during protocorm development. This study provides new insights into the regulation of glyoxylate cycle during early protocorm development of orchids.

## Materials and Methods

### Plant materials and growth conditions

Collection and growth of *P. aphrodite* seeds was as described by Balilashaki *et al*.^38^ with some modification. Mature seeds were collected from the capsules of *P. aphrodite*. Seeds were surface-sterilized with 1% (v/v) NaOCl solution for 15 min. After rinsing with sterilized water twice, seeds were re-suspended in half-strength Murashige and Skoog liquid medium to imbibe for 48 h, then applied to 0.85% (w/v) agar plates containing 1/2 MS salts with or without 1% (w/v) sucrose and grew in culture room at 23~25 °C. Protocorms were then collected at 0, 4, 7, 12, 20 and 30 days after incubation (DAI).

### Histology

To reveal the content in the *P. aphrodite* mature seeds, seeds were stained with Sundan IV, Sudan black and iodine for 40 min at 50 °C, and then washed with 70% ethanol. The stained sections were observed by microscopy.

### Transmission electron microscopy (TEM)

The *P. aphrodite* 0 DAI protocorms were prefixed in 4% (g/v) paraformaldehyde and 2.5% (v/v) glutaraldehyde in 67 mM phosphate buffer (NaH_2_PO_4_˙H_2_O and Na_2_HPO_4_) for 24 h. The samples were fixed in 1% (g/v) OsO4 in 0.067 M phosphate buffer, dehydrated through an acetone series (15–30–50–70–90–100%), embedded in Spurr’s resin and polymerized in a vacuum oven at 70 °C. Sections 100 nm thick were obtained and placed on grids, then counterstained with uranyl acetrate and lead nitrate, and observed by transmission electron microscopy (JEM-1400, JEOL).

### RNA preparation

Total RNA was extracted as described^29^. Plant materials were immersed in liquid nitrogen and stored at −80 °C. Briefly, frozen tissue (0.5–1 g) was pulverized with liquid nitrogen by using a pestle and mortar and then homogenized in TRIZOL reagent. The dissolved RNA was extracted with chloroform. After centrifugation at 13000 rpm to remove insoluble material, total RNA was precipitated with 0.8 M sodium citrate and 1.2 M NaCl at −20 °C overnight, then precipitated again with 4 M LiCl, pelleted, and washed. The final RNA precipitate was dissolved in a suitable volume of sterilized DEPC-treated water. To remove the DNA contamination, total RNA was treated with RNase-free DNase I.

### Expression analyses by RT-PCR and real-time quantitative RT-PCR

RNA was used as a template for cDNA synthesis with reverse transcriptase and the SuperScript II kit (Invitrogen). Transcripts of *PaMLS*, *PaICL*, and 8 candidate TF genes including *PaANT, PaHB5, PaMADS2, PaMYB4, PaPIF3, PaRAV1-1, PaWRKY18* and *PaWRKY71* were detected by RT-PCR and real-time quantitative PCR. The primer pairs are in Supplementary Table S1.

The methods of RT-PCR and real-time quantitative RT-PCR were as described^39^ with modification. The RT-PCR program was 94 °C for 5 min for denaturation, then 94 °C for 30 s, 72 °C for 30 s, and extension at 72 °C for 30 min. Annealing temperature and number of amplified cycles varied with different primer pairs (*PaICL* and *PaMLS*: 58 °C/25 cycles, candidate TF genes: 62 °C/33 cycles). The amplified products were analyzed on agarose gel and photographed. Only one amplified band with expected size was detected for each of *PaICL* and PaMLS (Supplementary Fig. S1).

For real-time quantitative RT-PCR analysis, the PCR program was incubation at 50 °C for 2 min, then 95 °C for 10 min, and thermal-cycling for 40 cycles (95 °C for 15 s and 60 °C for 1 min) by using the ABI 7500 Real-Time PCR instrument^39^. Triplicate experiments were performed for each sample. Sequencing Detection System v1.2.3 (Applied Biosystems) was adopted for data analysis.

### 5′ Rapid amplification of cDNA ends (5′ RACE) for *PaICL* and *PaMLS*

The 5′ RACE was performed as described^29^. Briefly, the full-length cDNAs were synthesized by extending the 5′ ends of cDNA by using the SMART RACE cDNA amplification kit (Clontech, Palo Alto, CA, USA). First-strand cDNAs were synthesized from 1 μg total RNA from *P. aphrodite* protocorms at 0 DAI following the manufacturer’s protocol. The cDNA containing the 5′ end for *PaICL* clones was obtained by PCR amplification with a 5′-specific universal primer (Clontech) and 3′ gene-specific primer sequences for *PaICL*, 5′-GCCCCGAGAATGAACTGGTGGTC-3′ and *PaMLS*, 5′-GTCCCTTCTTTTACCTCCCCAA-3′. The thermal cycling protocol was initial denaturation at 94 °C for 5 min, followed by 35 cycles at 94 °C for 30 s, 53 °C for 30 s and 72 °C for 30 s, and a final extension at 72 °C for 7 min. RACE-products were re-amplified with gene-specific nested primer sequences for *PaICL*, 5′- CCTCTTGCTGCTTGCGGTCGTGGTAG-3′ and *PaMLS*, 5′-GCTCCGTTCTCACTCTGCTGG-3′ and the nested universal primer provided in the RACE kit. The PCR protocol consisted of an initial denaturation at 94 °C for 5 min, followed by 35 cycles at 94 °C for 30 s, 53 °C for 30 s and 72 °C for 30 s and a final extension at 72 °C for 7 min. The PCR products were cloned into the pGEM-T Easy vector (Promega, Madison, WI, USA) and sequenced from both strands of six positive clones selected randomly.

### Sequence alignments and phylogenetic analysis

The full-length proteins sequences of 34 genes retrieved from GenBank were aligned by using Clustal W with the default parameters. The neighbor-joining phylogenetic tree was conducted in MEGA5^40^ with default settings. Bootstrap value was obtained by 1,000 replicate runs.

### Comparison of transcriptomic profiling by RNA sequencing

RNA from *P. aphrodite* protocorms at 4 and 7 DAI grown in medium containing1/2 MS plus 1% (w/v) sucrose were collected as described above. Both 2 μg RNA samples were treated with Dnase I, and then sequenced by using Solexa/Illumina RNA-seq (Illumina Hiseq. 2000 platform, BGI Tech Solutions). Before assembly, high-quality reads were obtained by removing adaptor sequences, and low-quality reads were filtered by using TRIMMOMATIC^41^ from raw reads. The resulting high-quality reads were de novo assembled and annotated as described^42^. Transcript abundance was normalized by using the fragments per kilobase per million mapped reads (FPKM) method^43^.

### Promoter analysis of *PaMLS*

A fragment of a 2000-bp upstream sequence of *PaMLS* was obtained from the corresponding genome sequence in the *P. equestris* genome^18^, and then analyzed by using PlantPAN^44^ (http://plantpan.mbc.nctu.edu.tw/) to predict putative TF binding sites on the *PaMLS* promoter.

Ten-fold up-regulated unigenes were screened by comparing gene expression of protocorm transcriptomes at 4 and 7 DAI. The selected unigenes were BLAST searched in the TF database of *Oryza sativa* subsp*. japonica* from PlantTFDB^45^ (http://planttfdb.cbi.pku.edu.cn/) to identify the putative TF genes. These putative unigenes were then BLAST searched in the database against putative TF genes binding on the *PaMLS* promoter.

### Construction of transformed fusions

Genomic DNA was extracted from floral buds of *P. aphrodite* as described^29^. The 1-kb *PaMLS* promoter fragments were amplified from genomic DNA of floral buds of *P. aphrodite* by PCR-amplification with EX Taq DNA polymerase (Takara) by using forward primers (*PaMLS*p_1000_5′_*BamH* I) and a reverse primer (*PaMLS*p_3′_*Nco* I) (Supplementary Table S1). The PCR products were cloned into the pGM-T vector (GeneMark), and then digested with the restriction endonucleases *BamH* I and *Nco* I to obtain the promoter fragments. The promoter fragments were cloned into the pJD301 vector that contains a firefly luciferase gene.

The *PaHB5* coding sequence was amplified by PCR from cDNA of protocorms of *P. aphrodite* at 4 DAI that were grown on medium plus sucrose by using primers for PaHB5_5′_*Xba* I and PaHB5_3′_*BamH* I (Supplementary Table S1). The PCR products were cloned into the pGM-T vector (GeneMark), then digested with restriction endonuclease *BamH* I and *Xba* I to obtain the *PaHB5* fragment. The fragment was cloned into the multiple cloning sites downstream of the CaMV 35 S promoter present in the pBI221 vector.

### Transient expression experiments and dual luciferase reporter assay

The methods were majorly following description of Chuang *et al*.^46^ with some modification. The *PaMLS* promoter plasmids, *PaHB5* plasmid, and RL2-pJD301 (relina luciferase, internal control) were isolated by using the High-Speed Plasmid Mini Kit (Geneaid) coated on gold particles 1.6 μm in diameter by co-precipitation. Before particle bombardment, each floral organ was separated from the full-opening flower, and then placed on a central core 2 cm in diameter on solid agar medium. These sections were bombarded by using a Modle Biolistic PDS-1000/He system (BioRad) at 1100 psi helium gas pressure, 28.5 inch-of-Hg vacuum and 9 cm target distance. After bombardment, floral buds were grown at room temperature for 18 to 20 hr to allow for expression of the luciferase protein. Luciferase activity in the transfected floral buds was measured by using the dual-luciferase reporter assay system (Promega). To prepare cell lysates, transfected floral buds were ground into fine powder by the addition of liquid nitrogen, and 1X phosphate buffered saline was added. The firefly (*Photinus pyralis*) luciferase reporter assay involved adding cell lysates to Luciferase Assay Reagent II. Then, firefly luciferase luminescence was quenched and renilla luciferase (internal control) was activated by adding Stop & GloR Reagent. The luciferase activity was measured with the TD-20/20 Luminometer system (BD Monolight 3010 C) with a 2-sec pre-measurement delay followed by a 10-sec measurement period for each assay. The relative luciferase activity was calculated as the ratio of firefly to renilla luciferase activity.

## Supplementary information

Supplementary information.
