# Supplementary material for: Expression regulation of MALATE SYNTHASE involved in glyoxylate cycle during protocorm development in Phalaenopsis aphrodite (Orchidaceae)
Source: Sci Rep. 2020 Jun 22;10:10123. doi: 10.1038/s41598-020-66932-8 (PMC7308390; doi:10.1038/s41598-020-66932-8)
Supplement: Supplementary file 1 — Supplementary information. [file 41598_2020_66932_MOESM1_ESM.pdf]

Supplementary Information

Expression regulation of malate synthase involved in glyoxylate cycle during protocorm development in *Phalaenopsis aphrodite* (Orchidaceae)

Wan-Lin Wu[1,2§], Yu-Yun Hsiao[3§], Hsiang-Chia Lu[1,2,4,] , Chieh-Kai Liang[2], Chih-Hsiung Fu[6], Tian-Hsiang Huang[3], Ming-Hsiang Chuang[5], Li-Jun Chen[1], Zhong-Jian Liu[4*], and Wen-Chieh Tsai[2,3,5,*]

Content

Supplementary Figures..3

Figure S1. Expression profiles analysis of *PaICL* and *PaMLS* of different developmental protocorm stage in *P. aphrodite* by RT-PCR..3

Figure S2. Number of unigenes related to transcription factors in each transcription factor family...4

Supplementary Tables..5

Table S1 List of primers used in this study...5

Table S2 Prdicted ATHB5 binding sites on 2000 bp upstream sequence of *PaMLS*......6

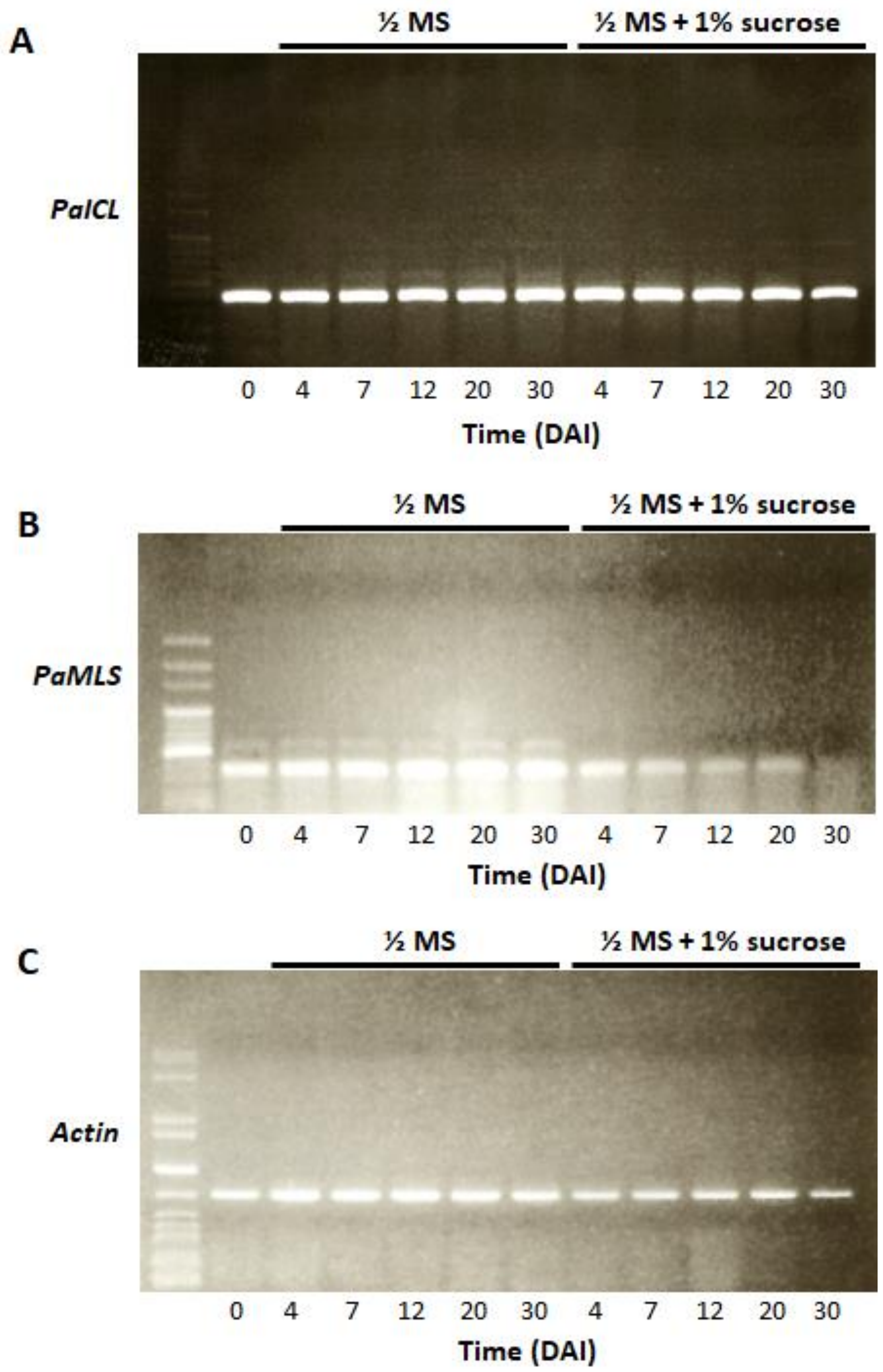

Figure S1. Expression profiles analysis of *PaICL* and *PaMLS* of different developmental protocorm stage in *P. aphrodite* by RT-PCR.

P. aphrodite seeds after resuspended in ½ MS liquid medium for 48 hr which were defined as 0 DAI protocorms. 4 to 30 DAI protocorms incubated in the absence of sucrose or in the presence of 1% sucrose of ½ MS medium. (A) PaICL (B) PaMLS (C) Actin.
(DAI, days after incubation).

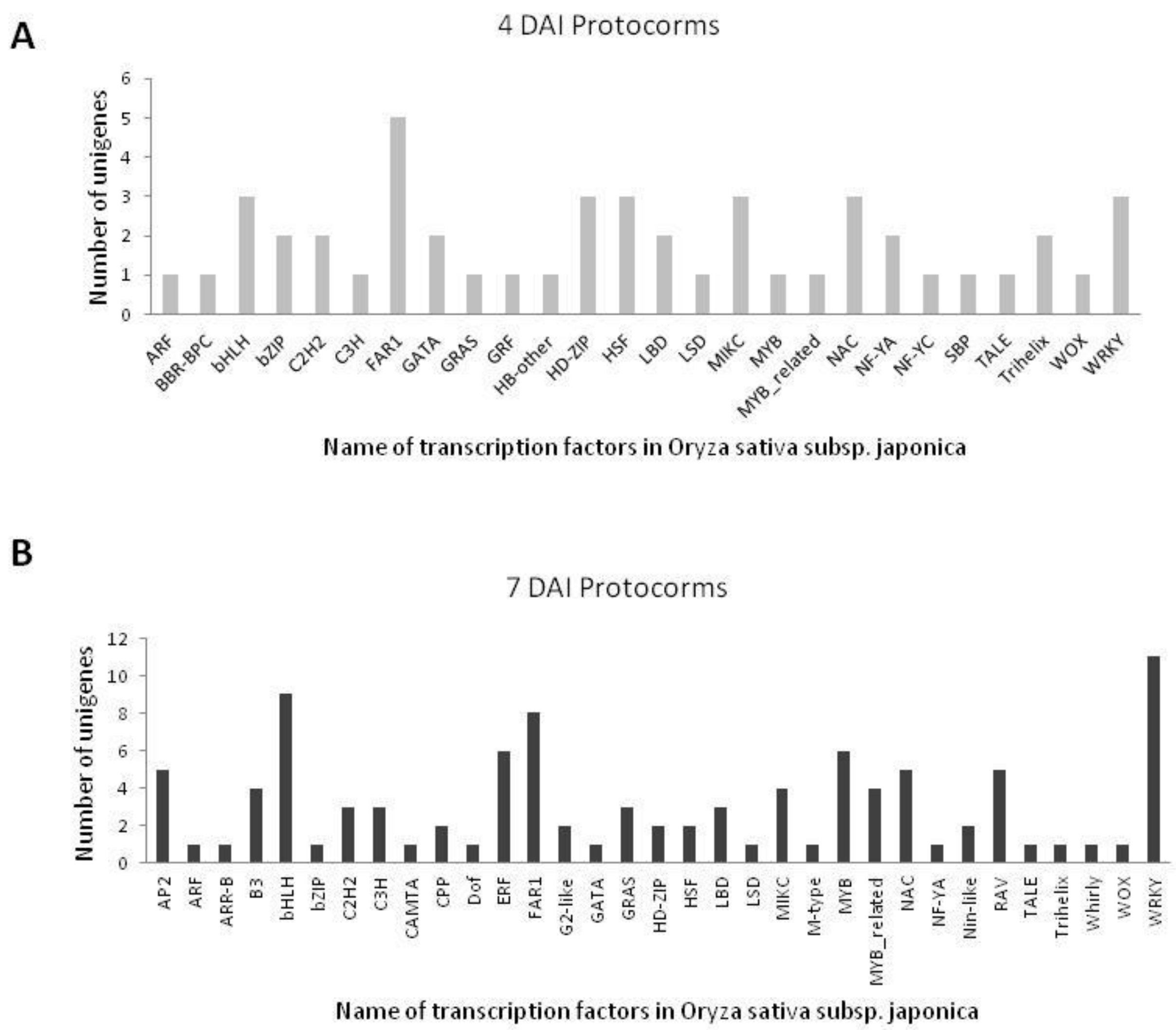

Figure S2. Number of unigenes related to transcription factors in each transcription factor family

Number of unigenes related to transcription factors 10-fold up-regulated in (A) 4-DAI protocorms and (B) 7-DAI protocorms.

Table S1 List of primers used in this study

Primer name	Sequence
**For real-time quantitative PCR**	
QPaMals-F	CGTGGAACAAGGTCGAGCTTTAGG
QPaMals-R	TGGGGAACAAGCTCCACAACCTTC
QPaICL-F	GCAAATCAGGTCCGGGACGATG
QPaICL-R	ATCCATCCACTCCTAAACATCGAATCC
QPaANT-F	AGTGGTGGAGTTGGAGGATTTG
QPaANT-R	GCATCGAGACTCCAAGCA
QPaHB5-F	GATGAGGGGGATGGAATGTATG
QPaHB5-R	CAGCTCCATATTTGTTATCACATTCC
QPaMADS2-F	AGGGAAACTTACCGCGCTCTA
QPaMADS2-R	GAGATGGGGGATCTATTCAGAGAGA
QPaMYB4-F	ATCGAGTGATGATGAGGCTTATTTT
QPaMYB4-R	AACCCAAGTCATCCAAACTCC
QPaPIF3-F	GCGTGTTGTTTGGCGCAAGTGGCAT
QPaPIF3-R	AGGCGCCCAAATATCAGCACCGTTG
QPaRAV1-1-F	CAGTTATGGGGATTCATGATTTTTC
QPaRAV1-1-R	TTCTCCGCATCCAAATCCATC
QPaWRKY18-F	GAAGAACTCCGATAACTTTGAACAGA
QPaWRKY18-R	AATGCAAATGAGTTCAAGGTCTAGT
QPaWRKY71-F_2	TCCCGCTTATCCGAAAACAT
QPaWRKY71-R	TCGAGCCGCAAGGTTCAA
**For RACE**	
3’PaMLS_F1	GTCCCTTCTTTTACCTCCCCAA
3’PaMLS_F2	GCTCCGTTCTCACTCTGCTGG
5’PaICL_R1	GCCCCGAGAATGAACTGGTGGTC
5’PaICL_R2	CCTCTTGCTGCTTGCGGTCGTGGTAG
**For *PaMLS* promoter-reporter plasmid construction**	
PaMLSp_1000_5’_*BamH* I	GGATCCTTCAGATAGGTTACAGGTAAATTATATAGA
PaMLSp_3’_*Nco* I	CCATGGCGTCACCACCGGAG
**For *PaHB5* plasmid construction**	
PaHB5_5’_*Xba* I	TCTAGAATGGAAGAAGAGAAGTCTCTGATCA
PaHB5_3’_*BamH* I	GGATCCTCATCCATCCCAAACTCCC

Table S2 Prdicted ATHB5 binding sites on 2000 bp upstream sequence of *PaMLS*

Putative binding site	Strand [a]	Putative binding sequence
-1662	+	ttcTTATTg
-1328	+	ggcTTATTa
-1108	+	aacTTATTt
-800	+	caaTTATTt
-796	+	tatTTATTt
-792	+	tatTTATTt
-769	-	aAATAAaaa
-756	-	aAATAAgaa
-709	+	tatTTATTa
-706	+	ttaTTATTt
-702	+	tatTTATTt
-611	+	taaTTATTt
-598	+	aagTTATTa
-590	-	aAATAAtat
-559	+	aaaTTATTt
-499	+	aatTTATTt
-487	-	tAATAAatt
-334	-	aAATAAata
-330	-	aAATAAtat
-296	-	aAATAAtat
-278	-	tAATAAaaa
-241	-	aAATAAcga

a: Forward or reverse directions of binding sequences
